# Ventricular Tachycardia and Resembling Acute Coronary Syndrome During Pheochromocytoma Crisis: A Case Report

**DOI:** 10.1097/MD.0000000000003297

**Published:** 2016-04-08

**Authors:** Shi-jun Li, Tao Wang, Lin Wang, Zhan-qi Pang, Ben Ma, Ya-wen Li, Jian Yang, He Dong

**Affiliations:** From the Department of Cardiology, Dalian Municipal Central Hospital Affillated of Dalian Medical University, Dalian, China.

## Abstract

Pheochromocytomas are neuroendocrine tumors, and its cardiac involvement may include transient myocardial dysfunction, acute coronary syndrome (ACS), and even ventricular arrhythmias.

A patient was referred for evaluation of stuttering chest pain, and his electrocardiogram showed T-wave inversion over leads V1 to V4. Coronary angiography showed 90% stenosis in the mid-left anterior descending coronary artery (LAD), which was stented. Five days later, the patient had ventricular tachycardia, and severe hypertension, remarkable blood pressure fluctuation between 224/76 and 70/50 mm Hg. The patient felt abdominal pain and his abdominal ultrasound showed suspicious right adrenal gland tumor. Enhanced computed tomography of adrenal gland conformed that there was a tumor in right adrenal gland accompanied by an upset level of aldosterone.

The tumor was removed by laparoscope, and the pathological examination showed pheochromocytoma. After the surgery, the blood pressure turned normal gradually. There was no T-wave inversion in lead V1-V4. Our case illustrates a rare pheochromocytoma presentation with a VT and resembling ACS. In our case, the serious stenosis in the mid of LAD could be explained by worsen the clinical course of myocardial ischemia or severe coronary vasospasm by the excessive amounts of catecholamines released from the tumor. Coronary vasospasm was possible because he had no classic coronary risk factors (e.g. family history and smoking habit, essential hypertension, hyperglycemia and abnormal serum lipoprotein, high body mass index). Thus, pheochromocytoma was missed until he revealed the association of his symptoms with abdominalgia.

As phaeochromocytomas that present with cardiovascular complications can be fatal, it is necessary to screen for the disease when patients present with symptoms indicating catecholamine excess.

## INTRODUCTION

Pheochromocytomas are neuroendocrine tumors that can present as sustained or paroxysmal hypertensive emergencies due to catecholamine excess. Transient myocardial dysfunction and acute coronary syndrome (ACS) are the most reported fatal cardiac events associated with catecholamine excess caused by pheochromocytomas.^1^ Sinus tachycardia, and even serious ventricular arrhythmia, are common arrhythmias that occur with phaeochromocytomas. We present a case of a rare pheochromocytoma presentation of ventricular tachycardia (VT) and resembling ACS. We obtained written consent from the patient to publish this report and did not include identifying patient data.

### Case Report

**FIGURE 1:**
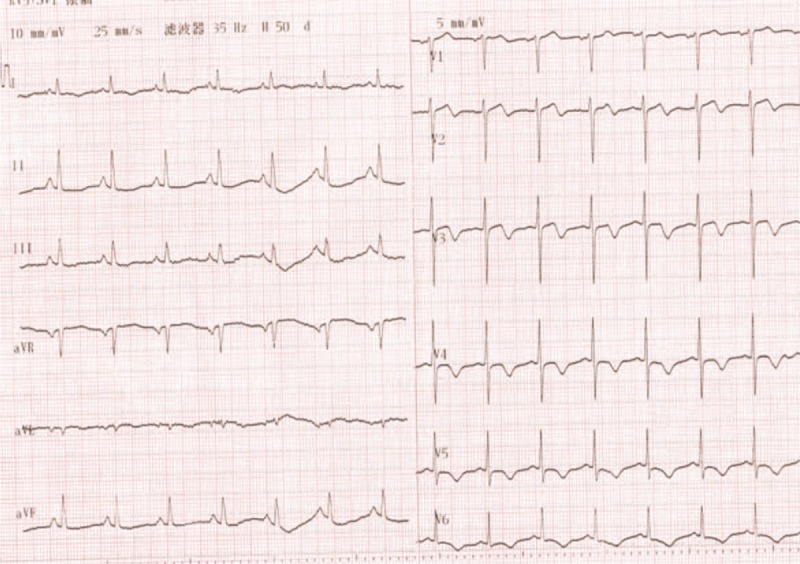
Electrocardiogram (ECG) in charge T-wave inversion over leads V1 to V4. ECG = electrocardiogram.

**FIGURE 2:**
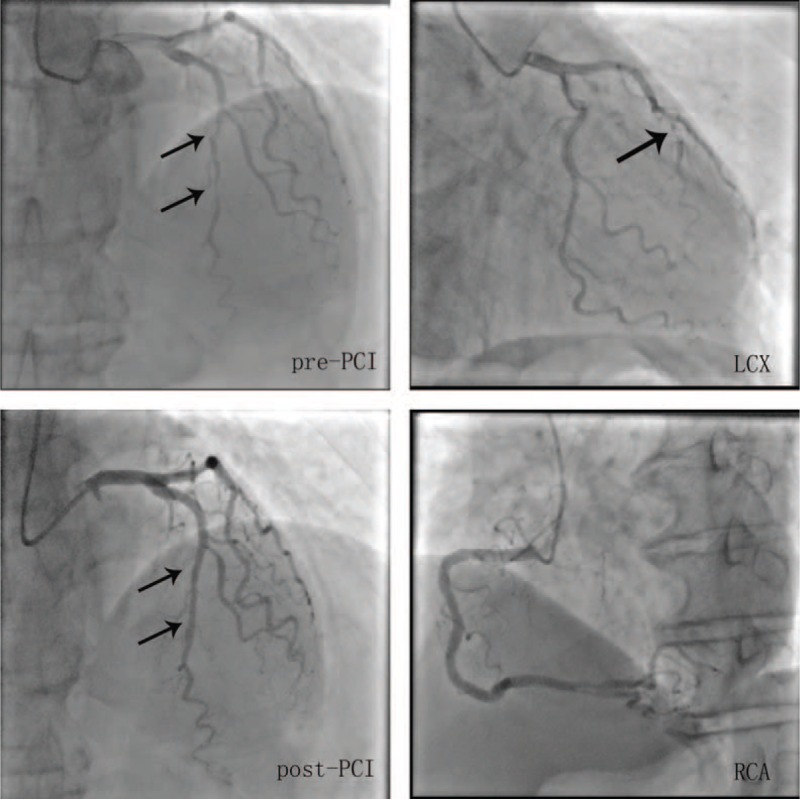
(A) Coronary angiography shows 90% stenosis in the mid-left anterior descending artery (LAD); (C) LAD was stented. LAD = left anterior descending.

**FIGURE 3:**
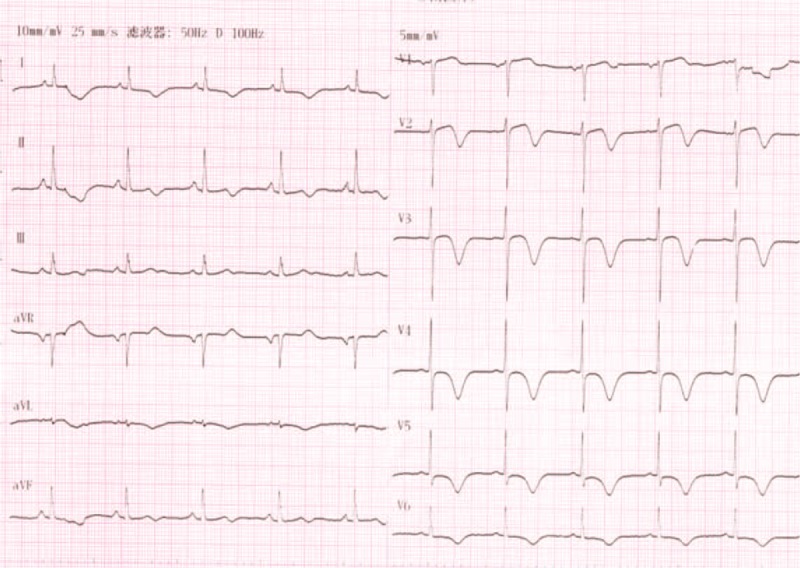
ECG after Coronary angiography T-wave still inversion over leads V1 to V4. ECG = electrocardiogram.

**FIGURE 4:**
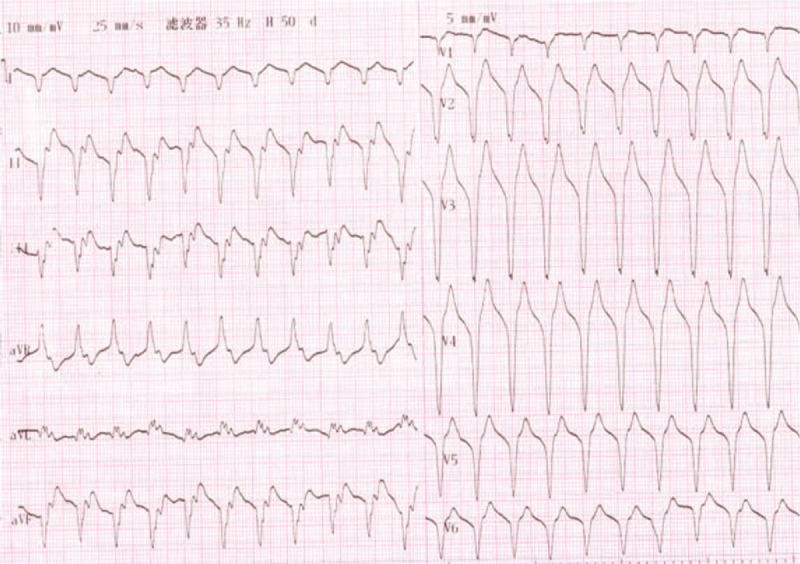
ECG showed VT. ECG = electrocardiogram, VT = ventricular tachycardia.

A 65-year-old man was referred to our hospital for evaluation of stuttering chest pain for 10 days, and he was a common worker. There was no positive finding from the relevant physical examination. He has no medical, family, and psychosocial history including co-morbidities, and relevant genetic information. His electrocardiogram showed T-wave inversion over leads V1 to V4 (Figure 1). Coronary angiography showed 90% stenosis in the mid-left anterior descending coronary artery (LAD), which was stented (Figure 2). T-wave still inversion over leads V1 to V4 after the percutaneous coronary intervention (PCI) (Figure 3). The patient discharged after the PCI in 5 days and recharged in the hospital because of a palpation. His electrocardiogram demonstrated ventricular tachycardia (Figure 4), and severe hypertension, remarkable blood pressure fluctuation between 224/76 and 70/50 mm Hg. Although several antihypertensive drugs were used, ventricular tachycardia still occurred on him for 2 times, each was preceded by a period of blood pressure fluctuation and burst out concomitantly at the peak of a hypertension crisis.

**FIGURE 5:**
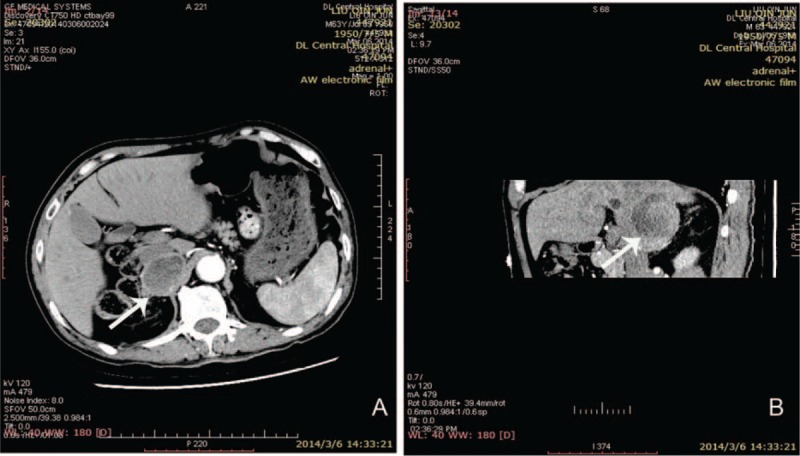
Abdominal computed tomography scan (arterial phase) a tumor in right adrenal gland accompanied.

**FIGURE 6:**
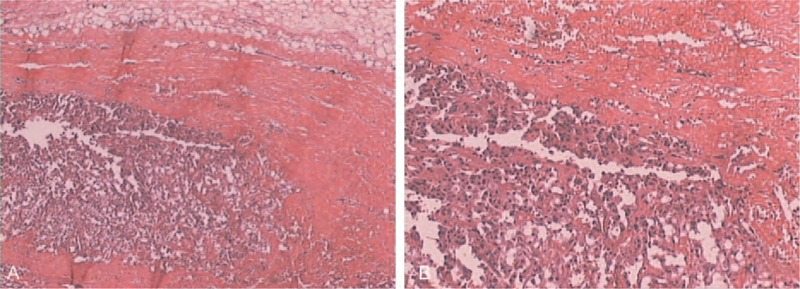
Pathological examination showed pheochromocytoma.

**FIGURE 7:**
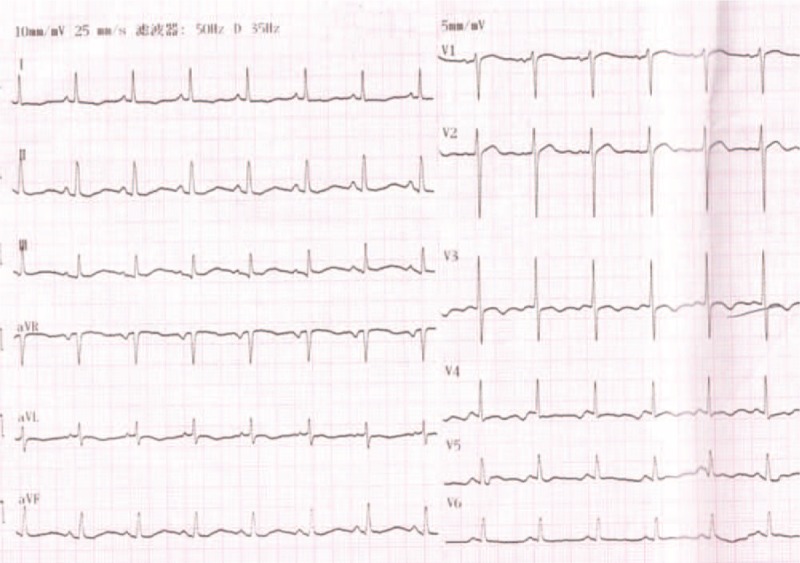
ECG after surgery (T-wave is normal). ECG = electrocardiogram.

The patient felt abdominal pain and his abdominal ultrasound showed suspicious right adrenal gland tumor. Enhanced computed tomography of adrenal gland conformed that there was a tumor in right adrenal gland accompanied by an upset level of aldosterone (Figure 5). The tumor was removed by laparoscope, and pathological examination showed pheochromocytoma (Figure 6). After the surgery, the blood pressure turned normal gradually. There was no T-wave inversion in lead V1-V4 (Figure 7).

## DISCUSSION

Our case illustrates a rare pheochromocytoma presentation with VT and resembling ACS. When the β-adrenoceptors are stimulated by catecholamine excess, it may lead to sinus tachycardia or even serious ventricular tachycardia.^2–7^ Takotsubo cardiomyopathy with transient myocardial dysfunction is a newly identified cardiac manifestation of phaeochromocytoma;^8^ severe coronary vasospasm may be the cause of some of the reported Takotsubo cardiomyopathy cases that have accompanied pheochromocytoma crises. Sometimes, after the removal of the tumor, the structural abnormalities and symptoms resolve. Cases involving pheochromocytoma crises resembling acute myocardial infarctions (AMI) have been reported in recent years.^9–12^ More than half of patients with pheochromocytoma crises presenting AMI and ACS had few significant coronary atherosclerosis; catecholamine excess lead to hemodynamic compromise of the myocardium that accelerated cell fibrosis and death. In patients with pheochromocytoma crises presenting AMI had significant coronary atherosclerosis, catecholamine excess may worsen the myocardial infarction. In our case, the occlusion in the mid of LAD could be explained by the worsening of the clinical course of myocardial ischemia or by severe coronary vasospasm caused by the excessive amounts of catecholamine released from the tumor. Coronary vasospasm appeared likely in this case because the patient had no classic coronary risk factors (e.g., family history, smoking, essential hypertension, hyperglycemia, abnormal serum lipoprotein, and high body mass index). Thus, pheochromocytoma was missed until he revealed the association of his symptoms with abdominalgia. As phaeochromocytomas that present with cardiovascular complications can be fatal, it is necessary to screen for the disease when patients present with symptoms, indicating catecholamine excess.
